# Examining the Disparities in Senior Center Built Environment

**DOI:** 10.1093/geroni/igaf122.2587

**Published:** 2025-12-31

**Authors:** Hui “Jimmy” Xie, Cinthia Camacho, Maryna Bankovska, Xiao Zhang, Bing Han, Deborah Cohen

**Affiliations:** California State University, Northridge, Northridge, California, United States; California State University, Northridge, Northridge, California, United States; California State University, Northridge, Northridge, California, United States; California State University, Northridge, Northridge, California, United States; Kaiser Permanente Southern California, Pasadena, California, United States; Kaiser Permanente Southern California, Pasadena, California, United States

## Abstract

Built environment has substantial influence on older adults’ health and wellbeing. Despite that, there is a scarcity of research on the built environment of senior centers, an important place for many older adults to socialize and engage in leisure activities. This study examined the disparities in the built environment of senior centers in 24 communities in the Greater Los Angeles Area. Each center was jointly assessed by 2–3 trained research assistants in 2023 using the Senior Center Assessment Tool (v1.0), covering the access, exterior, and interior of the center. Additionally, statistics about communities’ socioeconomic condition were retrieved from US Census Bureau database. On average, the study centers had 8.5 rooms (range: 1–19) with multi-purpose room being the most common type of room. Over half the centers had designated rooms/spaces for congregated meals, tabletop games and billiards, arts/craft, or computer use. The percentage of centers with an excellent overall condition of landscaping, exterior, and interior were 29%, 25%, and 35%, respectively. Results of bivariate correlations showed that community’s median household income had a positive relationship with overall condition of the interior (r=.42, p<.05), building exterior (r=.42, p<.05), and landscaping (r=.48, p<.05), percentage of rooms in excellent condition (r=.45, p<.05), and ADA compliance (r=.45, p<.05), and had a negative relationship with presence of safety issue(s) (r=-.57, p<.01). Substantive differences were found in the quality of built environment at senior centers across communities.

